# Host Responses to Respiratory Syncytial Virus Infection

**DOI:** 10.3390/v15101999

**Published:** 2023-09-26

**Authors:** Ayse Agac, Sophie M. Kolbe, Martin Ludlow, Albert D. M. E. Osterhaus, Robert Meineke, Guus F. Rimmelzwaan

**Affiliations:** Research Center for Emerging Infections and Zoonoses, University of Veterinary Medicine Hannover, 30559 Hannover, Germany; ayse.agac@tiho-hannover.de (A.A.); sophie.madleine.kolbe@tiho-hannover.de (S.M.K.); martin.ludlow@tiho-hannover.de (M.L.); albert.osterhaus@tiho-hannover.de (A.D.M.E.O.); robert.meineke@tiho-hannover.de (R.M.)

**Keywords:** respiratory syncytial virus, innate immunity, adaptive immunity, immunopathology, immune evasion, vaccines

## Abstract

Respiratory syncytial virus (RSV) infections are a constant public health problem, especially in infants and older adults. Virtually all children will have been infected with RSV by the age of two, and reinfections are common throughout life. Since antigenic variation, which is frequently observed among other respiratory viruses such as SARS-CoV-2 or influenza viruses, can only be observed for RSV to a limited extent, reinfections may result from short-term or incomplete immunity. After decades of research, two RSV vaccines were approved to prevent lower respiratory tract infections in older adults. Recently, the FDA approved a vaccine for active vaccination of pregnant women to prevent severe RSV disease in infants during their first RSV season. This review focuses on the host response to RSV infections mediated by epithelial cells as the first physical barrier, followed by responses of the innate and adaptive immune systems. We address possible RSV-mediated immunomodulatory and pathogenic mechanisms during infections and discuss the current vaccine candidates and alternative treatment options.

## 1. Introduction

Respiratory syncytial virus (RSV) is a widely circulating pathogen in the human population and a major cause of acute lower respiratory tract infections (ALRIs) in infants and older adults. RSV is an enveloped, negative-sense, single-stranded RNA virus belonging to the *Orthopneumovirus* genus of the family *Pneumoviridae* [1]. The viral genome consists of ten genes encoding eleven proteins: nonstructural proteins 1 and 2 (NS1/2), nucleocapsid protein (N), matrix protein (M), phosphoprotein (P), small hydrophobic protein (SH), glycoprotein (G), fusion protein (F), large protein (L), and M2 with two overlapping open-reading frames leading to two proteins, M2.1 and M2.2 [1]. Two antigenic subtypes, RSV-A and -B, are distinguished, each with several genotypes [2,3]. Currently, genotypes ON1 (RSV-A) and BA-CC (RSV-B) are circulating and differ from previous genotypes by the presence of sequence duplications of 72 bp and 60 bp, respectively, in the second hypervariable region of the G gene [4]. Both subtypes may co-circulate during an outbreak, with RSV A being the predominant strain in most years, although regional and seasonal differences are common [4].

RSV infections are a major cause of severe ALRI in infants and older adults [5,6,7,8]. In 2019, approx. 33 million RSV cases in infants were reported, of which 3.2 million required hospitalization [9]. Children under six months of age are at higher risk of hospitalization and fatal outcomes, accounting for ~50% of total hospitalized ALRI cases in high-income countries [9]. In older adults, 5.2 million RSV cases of ALRI were reported in the same year, resulting in 470,000 reported hospitalizations and 33,000 in-hospital deaths [10]. During the SARS-CoV-2 pandemic, reduced activity of various respiratory viruses, including RSV, was reported [11,12], probably due to non-pharmaceutical preventive measures to limit the transmission of SARS-CoV-2. Subsequently, increased sizes and numbers of outbreaks, with different times of onset in comparison to previous years, were reported for RSV disease, mainly affecting infants and children [12]. The decrease in RSV infections during the pandemic may have reduced immunity in children, referred to as the ‘immunity dept’, making them more susceptible to severe infections [12,13]. 

Nearly all infants are infected with RSV by the age of 2 years [14]. Reinfections occur throughout life, while disease severity tends to diminish with subsequent exposures. Older adults, however, represent a population that is also at risk of more severe disease during RSV infection [15,16,17]. Most infections in infants are mild, leading to an upper respiratory tract illness (URTI) or ‘flu-like’ symptoms often accompanied by otitis media [18,19]. Severe ALRI, mainly observed in infants younger than six months of age, can lead to bronchiolitis, pneumonia, and croup [18,19]. ALRIs are characterized by rhinorrhea, dry/wheezy cough, tachypnea, and dyspnea [19]. Infants, the immunocompromised, and older adults have a higher risk of developing severe infections [5,6,7,20,21]. Several studies further indicated a correlation between RSV-associated hospitalization during infancy and the development of asthma and recurrent wheezing later in life [22,23,24,25,26]. However, other factors, like comorbidities, may also account for this [6,27,28].

Virus-neutralizing human monoclonal antibodies directed to the F protein, i.e., Synagis^®^ (Palivizumab) and Beyfortus^®^ (Nirsevimab), have proven effective in lowering the risk of ALRI in high-risk infants when administered prophylactically. However, vaccine development for RSV has been challenging. The first clinical trials in infants in the 1960s with a formalin-inactivated RSV vaccine (FI-RSV) led to enhanced respiratory disease (ERD) in infants following their first natural infection [29]. Since then, vaccine development has been approached more cautiously while research has focused on understanding the immune response and immunopathogenesis during infection. The immaturity of an infant’s immune system as well as immunosenescence and preexisting immunity in older adults represent the most significant challenges for the development of a protective vaccine [30]. Several promising RSV vaccine candidates based on various production platforms have been developed and are currently in clinical trials. Recently, two protein-based vaccines, Arexvy (GlaxoSmithKline Biologicals (GSK), Brentford, UK) and Abrysvo (Pfizer, New York, NY, USA), were approved for use in older adults. Furthermore, a prefusion F-protein-based RSV vaccine candidate was developed for use in pregnant women to protect their infants early in life and was recently approved by the FDA. 

Previous work illustrates the importance of understanding the correlation between protective immunity and immunopathogenesis in severe cases of RSV infections for the development of efficient and protective vaccines and therapeutics for those most at risk. This review focuses on the current knowledge of host immune responses to RSV infection, leading to either viral control or more severe disease due to immunopathogenesis, and the current use of this knowledge in vaccine development.

## 2. Innate Immune Responses to RSV Infection 

**Figure 1:**
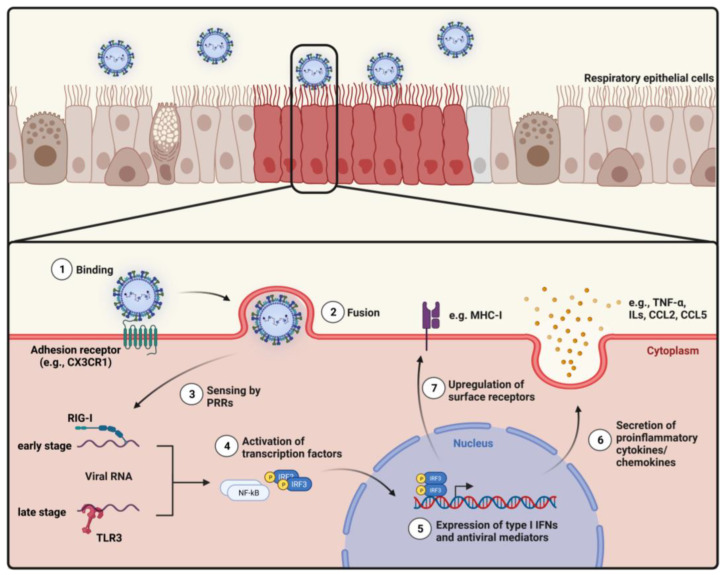
Overview of the intracellular mechanisms in airway epithelial cells (AECs) after respiratory syncytial virus (RSV) entry. RSV enters ciliated AECs by binding to receptors on the surface of the cells (e.g., CX3CR1), followed by internalization of the virus particle into the cytoplasm. Following infection, intracellular pattern recognition receptors, like RIG-I or TLR3, sense viral ssRNA or dsRNA intermediates resulting in the activation of transcription factors like NK-κB or IRF3. Nuclear translocation of transcription factors leads to the expression of several antiviral mediators, like cytokines, chemokines, and receptors for antigen presentation. RIG-I: retinoic-acid-inducible protein I; TLR3: toll-like receptor 3; IRF3: interferon regulatory factor 3; IFN: interferon; TNF-α: tumor necrosis factor α; IL: interleukin; MHC-I: major histocompatibility complex I. Created with BioRender.com.

Although the innate immune response toward infection with different *Pneumoviridae* members is still far from clear and may differ considerably [31,32,33,34], for RSV infection of humans many different mechanisms have been described. Epithelial cells are the primary target of infection (Figure 1) and secrete proinflammatory mediators upon RSV infection, leading to the recruitment and activation of innate immune cells. Several cell types are involved in the innate immune response, such as polymorphonuclear cells, cytotoxic lymphocytes, i.e., natural killer (NK) cells, or mononuclear phagocytes [35,36]. Tissue-resident macrophages and dendritic cells are among the first innate immune cells activated and mediate the further recruitment of inflammatory leukocytes and lymphocytes, like eosinophils, neutrophils, monocytes, and NK cells, to the site of infection by the secretion of type I IFNs and chemokines [37]. These cells subsequently begin eliminating the virus and simultaneously mediate the activation and recruitment of adaptive immune cells [36]. The initial immune response during epithelial cell infection, the protective role of innate immune cells, and their potential contribution to immunopathogenesis during RSV infections will be discussed in the following sections.

### 2.1. Infection of Epithelial Cells

The airway epithelium and associated mucus represent the first physiochemical barrier during respiratory infections [38]. The pseudostratified epithelium consists of three major cell types, namely ciliated cells, basal cells, and non-ciliated, secretory goblet cells which are connected by tight junctions [39,40]. The epithelial cells are mainly covered by mucus, contributing to protection from inhaled pathogens. The mucus is comprised of two distinct layers: a periciliary liquid layer, allowing the movement of cilia, and a more viscous upper layer, immobilizing the pathogen. Both layers consist of mucins, defensins, lysozymes, and immunoglobulins [41]. Mucociliary clearance, resulting from the continuous beating of motile cilia and consequently the movement of the outer mucus layer toward the pharynx, forms the first innate defense mechanism against respiratory pathogens. The protective properties of the mucus layer have already been shown for various respiratory viruses, like influenza viruses or coronaviruses [42,43,44,45]. An increased susceptibility of older adults to respiratory infections may be partly explained by a decrease in mucus production, cilia movement, and mucociliary clearance resulting in reduced pathogen removal [42,46].

Once RSV has traversed the mucus layer, it primarily infects airway epithelial cells (AECs), usually via the apical surface [47]. The infection of AECs depends on the cell’s differentiation status as infection increases with ciliogenesis. RSV therefore preferentially infects polarized ciliated AECs resulting in a focal distribution of the infection [48]. Hallmarks of AEC infection are cell sloughing, cilia loss, mucus hypersecretion, and syncytia formation. The loss of cilia and increased mucus production may contribute to airway obstruction during infection [38,49].

Several viral receptors have been proposed, as reviewed in [50]. Among others, nucleolin [51,52], CX3CR1 [53,54,55,56], heparan sulfate proteoglycans (HSPGs) [57,58,59], and intracellular adhesion molecule-1 (ICAM-1) [60] were associated with RSV attachment [50]. The interaction of RSV F and G with the HSPGs on the surface of immortalized cell lines leads to viral attachment and facilitates infection [59]. However, since HSPGs are barely expressed on the apical surface of epithelial cells *in vivo*, other receptors may be required for RSV infection [59]. 

CX3CR1 is expressed on various cell types, including innate and adaptive immune cells, and on motile cilia of AECs, which matches the RSV cell tropism during an infection [56,61,62]. Only two ligands are known for CX3CR1: the RSV G protein and CX3CL1 (fractalkine) [61]. CX3CL1 is a transmembrane protein on the surface of epithelial cells and contributes to the cell adhesion of leukocytes during infection [61,63]. Soluble CX3CL1 serves as a chemoattractant for T cells and monocytes via interaction with CX3CR1 on the surface of these cells [64]. RSV G contains a CX3C motif in its conserved central region, implicating a direct interaction between RSV G and CX3CR1 [65]. A study by Ha et al. showed a possible interaction between the receptor and G since a mutation in the CX3C motif (CX3C to CX4C) reduced the infection of epithelial cells in RSV-infected cotton rats [66]. However, a direct biochemical interaction between the RSV G protein and CX3CR1 has not been demonstrated yet. These data show that although G may facilitate the infection of epithelial cells, it is not strictly necessary for it [66]. However, the direct interaction of G and CX3CR1 may not only affect the infection of AECs but also interfere with the CX3CR1-CX3CL1 interaction. Soluble G may thereby act as a chemoattractant resulting in the increased recruitment of CX3CR1^+^ immune cells to the site of infection. 

With the infection of respiratory epithelial cells, antiviral and innate immune responses are initiated. The response of AECs to RSV infection is similar to that against other viral infections like influenza viruses, SARS-CoV-2, or herpesviruses [67,68,69,70,71]. Upon viral attachment and entrance, intra- and extracellular pattern recognition receptors (PRRs) recognize the pathogen and initiate a signaling cascade resulting in the expression of antiviral genes [72]. Among the PRRs, retinoic-acid-inducible gene I (RIG-I)-like receptors (RLRs) and toll-like receptors (TLRs) were shown to play a role in virus recognition [73]. During the early stage of infection, RIG-I senses the viral RNA and activates the transcription factor interferon regulatory factor (IRF)-3, ultimately leading to the upregulation of MHC-I, NLRC5, and IFN-β [73]. During the later stages of an active infection, intracellular TLR3 binds to the viral RNA [73] and activates the transcription factor NF-κB, which induces the expression of type I interferons (IFN) [74,75]. Type I IFN signaling modulates metabolic pathways, thereby setting the cells in an ‘antiviral state’ that restricts viral replication and spreading to uninfected cells. Infection of AECs with RSV consequently induces an altered expression and secretion of chemoattractants and adhesion molecules, e.g., tumor necrosis factor (TNF)-α, CXCL6, CXCL10, RANTES/CCL5, interleukin (IL) 1β, IL-6, IL-8, CCL2, macrophage inflammatory protein 1α (MIP-1α), granulocyte colony-stimulating factor (G-CSF), granulocyte-macrophage colony-stimulating factor (GM-CSF), ICAM-1, vascular cell adhesion protein 1 (VCAM-1), and major histocompatibility complex I/II (MHC-I/II) [76,77,78,79,80,81,82,83]. Subsequently, their secretion triggers the recruitment of innate and adaptive immune cells and their interaction with infected AECs. 

The immune modulatory mechanisms of different RSV proteins are well-known and have been reviewed previously [84]. NS1 and NS2 play a significant role in immune modulation by interacting with components of different immune signaling pathways. For example, NS1/2 interferes with the Janus kinase (JAK)/signal transducer and activator of transcription (STAT) signaling by suppressor of cytokine signaling proteins (SOCS)-dependent inhibition of JAK kinases and induction of proteasome-mediated STAT degradation [85,86]. *In vitro* studies have also shown the capacity of NS1 to translocate into the nucleus, where it interacts with the mediator complex and chromatin, indicating a regulatory effect of NS1 on the host’s gene expression [87]. Further, the respective interactions of NS1 and NS2 with mitochondrial antiviral-signaling protein (MAVS) and RIG-I interfere with the activation of IRF3 and NF-κB [87,88,89,90]. IFN-β synthesis is impaired by RSV infection, and the expression of anti-apoptotic genes is increased [90,91,92]. Following this, previous *in vivo* and *in vitro* studies have shown that the modulation of immune responses by NS1/2 inhibited apoptosis but induced necroptosis in RSV-infected AECs, which is considered an inflammatory cell death variant [92,93,94]. The release of HMGB1 into the extracellular space during necroptosis causes the recruitment of proinflammatory and Th2-type cytokines, ultimately resulting in increased airway inflammation and disease severity [94]. Besides NS1/2, an immunomodulatory role was also proposed for RSV nucleoprotein (N). *In vitro* studies indicate that RSV N induces the formation of inclusion bodies that serve as ‘traps’ for MAVS and melanoma-differentiation-associated gene 5 (MDA-5) during an early stage of infection [95,96]. RSV N interacts with PKR leading to reduced phosphorylation of eIFα and reduction in PKR-mediated signaling [97]. The activity of RSV proteins in infected AECs not only interferes with intrinsic immune pathways but restricts the activation of several genes that are involved in the production of cytokines and chemokines, which are crucial for the recruitment of innate and adaptive immune cells [84]. 

AECs, thereby, initiate the first antiviral, innate immune response upon RSV infection by the increased expression of adhesion molecules on the surface and the secretion of a variety of cytokines and chemokines that are essential for the recruitment of innate and adaptive immune cells. However, RSV has developed ways to alter and suppress this initial immune response, possibly affecting the activation and recruitment of immune cells during the early phase of infection. As mentioned above, cilia movement and mucus production are important during the first encounter with RSV and other respiratory viruses. However, most *in vitro* models are not able to represent the airway epithelium in its physiological three-dimensional structure, cellular composition, and motility [98]. Air–liquid interfaces and organoids represent promising systems to investigate virus–host interactions and immune responses during an infection under more physiological conditions, when compared to immortalized cell lines, and are therefore of increasing importance for investigating respiratory viruses [99,100,101]. 

### 2.2. Eosinophils

Eosinophils are a subset of granulocytic, polymorphonuclear leukocytes originating in the bone marrow and can be detected in low numbers in the peripheral blood after maturation [102,103]. They can migrate to different tissues, where they contribute to the physiology of the tissue under homeostatic conditions [102,103]. Increased numbers of eosinophils and their recruitment to the infected tissue can be observed during viral infection, mediating the primary antiviral host defense through their phagocytic and antigen-presenting activity. The protective role of eosinophils during infection has been shown for several viruses, like Influenza A virus (IAV), SARS-CoV-2, human parainfluenza virus, and human rhinovirus [104,105,106,107]. 

**Figure 2:**
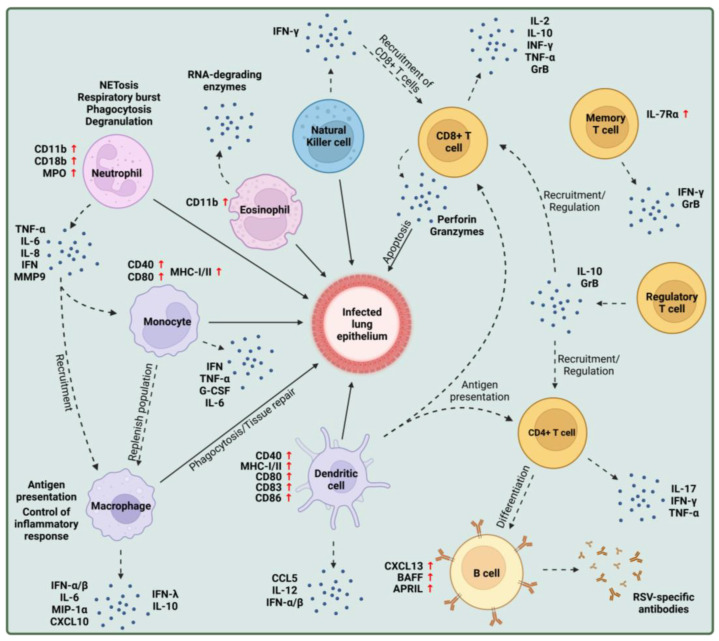
Schematic representation of the antiviral responses of innate and adaptive immune cells during RSV infection. Innate immune cells mediate the control of infection by direct interaction with the infected AECs, followed by their phagocytosis, or by direct interaction with virus particles, leading to the neutralization of the virus, e.g., by NETosis. Non-professional and professional antigen-presenting cells are responsible for the activation and recruitment of adaptive immune cells to the site of infection to further drive viral clearance. Regulation of immune responses is thereby crucial to avoid exaggerated, potentially immunopathogenic responses. IL: interleukin; IFN: interferon; TNF: tumor necrosis factor; GrB: granzyme B; BAFF: B cell-activating factor; APRIL: A proliferation-inducing ligand; MHC: major histocompatibility complex; MMP9: matrix metalloprotease 9. Created with BioRender.com.

Chemoattractants like CCL5 or MIP-1α, secreted by RSV-infected AECs, lead to the recruitment of eosinophils to the site of infection [108,109,110]. Following recruitment, eosinophils sense viral ssRNA in a TLR7-dependent manner [111]. This induces a signaling cascade resulting in the increased expression of the transcription factor IRF7 and, consequently, the increased expression of type I IFNs like IFN-β [111]. Upon interaction with infected AECs and viral RNA, eosinophils are activated, characterized by the increased surface expression of phagocytic surface marker CD11b (Figure 2) [111,112,113]. Activated eosinophils contribute to viral clearance by degranulation, defined by the secretion of RNA-degrading enzymes, such as the eosinophilic cationic protein (ECP) [109,114]. 

Despite their potential role in virus elimination, in the context of RSV, eosinophils are mostly known for their alleged role in ERD in infants following FI-RSV vaccination. Early studies with the tissue of the two fatal cases (*ex vivo*) reported an increased infiltration of eosinophils into the lungs compared to control groups [29]. Eosinophilia was consequently considered a hallmark of ERD. In the following years, several studies indicated that eosinophils do not directly contribute to ERD [115]. Vaccination studies with FI-RSV followed by RSV challenge in cotton rats demonstrated that levels of neutrophils and lymphocytes were increased in vaccinated rats compared to unimmunized control rats [116]. In mice depleted of regulatory T cells (Tregs), RSV infection led to increased disease severity and increased levels of eosinophils, CD4^+^, and CD8^+^ T cells in the bronchoalveolar lavage (BAL) [117]. CD4^+^ T cells found in the BALs of these mice had a Th2-type phenotype and secreted IL-13 during infection [117]. Therefore, excessive recruitment of eosinophilia may only result from an unbalanced Th2-biased T-cell response and the increased expression of Th2-type cytokines like IL-4, IL-10, and IL-13 [118]. 

**Figure 3:**
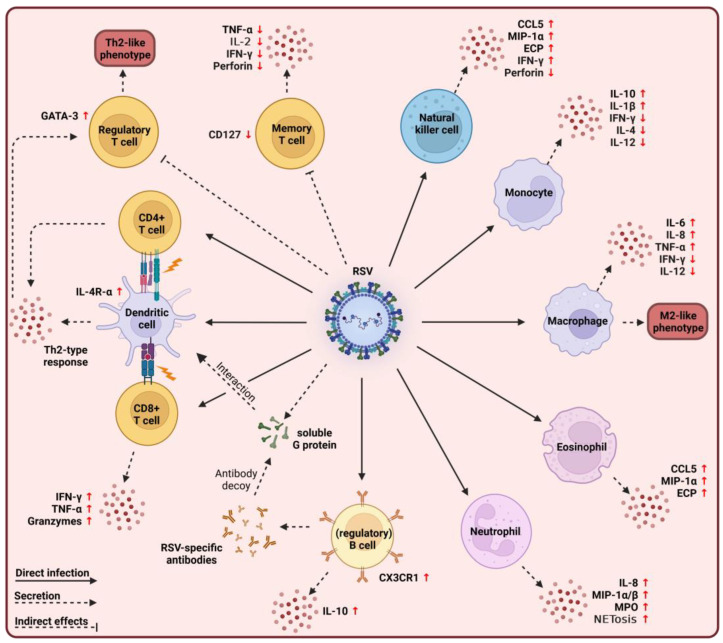
Overview of immunopathogenic responses mediated by innate and adaptive immune cells during RSV infection. Changes in the innate and adaptive immune response can be a consequence of microenvironmental changes, genetic predispositions, or interaction with the virus. RSV can directly infect a variety of immune cells or indirectly alter their immune response, e.g., by the secretion of soluble G, characterized by phenotype changes in immune cells, or the excessive secretion of immunosuppressive or proinflammatory cytokines. TNF-α: tumor necrosis factor α; IFN: interferon; IL: interleukin; ECP: eosinophil cationic protein; MIP-1α: macrophage inflammatory protein-1α; MPO: myeloperoxidase. Created with BioRender.com.

Apart from their still tentative role in FI-RSV-associated ERD, several *in vitro* and *ex vivo* studies suggested a contribution of eosinophils in severe cases of RSV infection (Figure 3). In RSV-infected infants, increased levels of ECP were detected in nasopharyngeal secretions that correlated with increased disease severity compared to uninfected controls [110,119]. The direct interaction or infection of eosinophils by RSV may explain their altered immune response as activated eosinophils secrete proinflammatory cytokines like CCL5, IL-6, and MIP-1α after viral interaction resulting in a proinflammatory milieu and increased influx of proinflammatory cells [79,120,121,122]. In IL-5-deficient mice, eosinophilic infiltration into the lungs was abolished following RSV infection and restored after IL-5 transfer into the mice, indicating a crucial role of IL-5 in eosinophil recruitment [123]. However, eosinophilic recruitment to the site of infection led to airway hyperresponsiveness and increased disease severity compared to sham-infected controls [123]. A recent study showed that infection of neonatal mice with RSV resulted in airway hyperresponsiveness and increased numbers of eosinophils, macrophages, and CD4^+^ T cells in the lungs compared to mock-infected mice, demonstrating that recruited eosinophils can contribute to disease severity [124]. 

Together, these studies indicate that excessive eosinophil recruitment to the lungs and subsequent lung pathology may result from altered CD4^+^ T cell signaling. Eosinophils usually contribute to protection against RSV infections. Th2-biased responses, however, lead to their increased recruitment and activation during infection and result in excessive and proinflammatory immune responses.

### 2.3. Neutrophils 

Neutrophils belong to the polymorphonuclear leukocyte system and constitute the most abundant leukocyte in the blood [125]. Similar to eosinophils, neutrophils can migrate into tissue during a steady state and contribute to tissue homeostasis [125]. Under pathological conditions, like viral infections, neutrophils are involved in virus detection and elimination, cytokine production, recruitment of proinflammatory immune cells, and antigen presentation [126,127]. Neutrophil activity is mainly based on three central mechanisms: phagocytosis, degranulation, and NETosis [126]. 

During RSV infection, chemoattractants like ICAM-1 and IL-8 produced by epithelial cells lead to the recruitment of neutrophils to the site of infection [75,128]. Following recruitment, neutrophils interact with ICAM-1 on the surface of RSV-infected AECs leading to their activation characterized by increased expression of CD11b, CD18b, and myeloperoxidase (MPO) (Figure 2) [129,130]. Once activated, neutrophils can initiate several mechanisms to inhibit the further spread of infection. The formation of neutrophil extracellular traps (NETs) is a hallmark of neutrophil activation. NETs are extracellular structures of histones, elastase, MPO, and defensins [126]. Viral particles are captured by these structures and neutralized by MPO and defensin secretion [126]. It was shown that the interaction of RSV F protein with neutrophilic bactericidal/permeability-increasing protein (BPI) induces NET formation in a TLR4- and reactive oxygen species (ROS)-dependent manner, thus promoting viral neutralization [131,132]. Neutrophils can further limit viral infection and replication by producing ROS (‘respiratory burst’), virus phagocytosis, and degranulation [129]. Neutrophils can release a subset of chemokines, cytokines, and antimicrobial substances like TNF-α, IL-6, IL-8, IFN, and matrix metalloprotease 9 (MMP9), resulting in the recruitment of monocytes and macrophages [133]. Neutrophils, therefore, can contribute to viral clearance during RSV infections by exhibiting an antiviral response and coordinating the immune signaling of innate and adaptive immune cells. 

An immunopathogenic role during RSV infection has also been proposed for neutrophils since their numbers were increased in the airways of infants with severe disease [134,135]. Enhanced interaction of neutrophils with AECs may induce excessive cell damage characterized by lower ciliary activity, cilium loss, and detachment [133,136]. Recent studies further implicated pulmonary neutrophilia as contributing to ERD following FI-RSV vaccination [137]. Direct infection of neutrophils led to the secretion of IL-8, MIP-1α/β, and MPO, resulting in an increased proinflammatory milieu (Figure 3) [138,139]. The increased secretion of IL-8, a potent chemoattractant for neutrophils, by infected neutrophils may lead to further recruitment of neutrophils to the infection site, contributing to immune pathogenesis [139]. Although NETs generally have an antiviral role, their disproportional formation during infections might enhance disease. Increased generation and accumulation of NETs were shown to block the airways of infants with severe infections [132,140]. In a recent experimental human infection study, the inflammatory activity of neutrophils in the respiratory mucosa of volunteers before infection predisposed the latter to symptomatic RSV infection [141]. The presence and activity of neutrophils were linked with impaired antiviral immune responses. Asymptomatic outcomes were characterized by IL-17 expression in the early phase of infection [141]. Preexisting neutrophilic inflammation, therefore, may counteract the protective response during the early stages of infection and instead lead to a higher risk of symptomatic infection [141]. 

Similar to other immune cells, neutrophils exhibit a bivalent role during RSV infections where time and microenvironmental conditions are critical for neutrophilic activity. Neutrophils may contribute to viral clearance by inhibiting viral spread and coordinating innate and adaptive immune responses. However, excessive neutrophil recruitment and activation of neutrophils can lead to an exaggerated immune response, correlated with disease severity in infants.

### 2.4. Natural Killer Cells

Bone-marrow-derived NK cells are granular lymphocytes best known for their cytotoxic activity [142]. Since NK cells do not undergo somatic hypermutation like other lymphocytes (i.e., B and T cells), they are considered part of the innate immune response [143]. NK cells are involved in various processes, like maintaining immune homeostasis, clearing tumors or infections, and regulating immune responses by interaction with different immune cells like T cells, macrophages, or dendritic cells [142,144]. NK cells contribute to viral clearance through the secretion of IFN-γ and the lysis of infected cells by perforin- and granzyme-dependent antibody-dependent cellular cytotoxicity (ADCC) [144].

Following RSV infection, NK cells accumulate in the lung and are an important source of IFN-γ, especially during the early phase of infection [145]. Besides their cytotoxic role, NK cells are involved in the IFN-γ-mediated recruitment of CD8^+^ T cells during infection (Figure 2) [145]. In RSV-infected mice, NK-derived IFN-γ prevented the development of lung eosinophilia in an IL-12-dependent manner [146]. Infection experiments in NK-deficient mice further showed an impaired IFN-γ production resulting in a Th2-skewed immune response and lung disease [147]. In secretions of RSV-infected infants with bronchiolitis, low numbers of NK cells and IFN-γ were detected, indicating that impaired NK activity is associated with more severe disease manifestations [148]. These studies thereby show a cytotoxic and immunoregulatory role of NK cells during RSV infection, mainly by the production of IFN-γ. In TLR4-deficient mice, the recruitment into the lungs and cytotoxic activity of NK cells was diminished upon infection compared to wild-type mice, indicating that RSV F protein activation of TLR4 is important for the recruitment and activity of NK cells [149].

Excessive recruitment of immune cells into the lungs during RSV infection, as seen for eosinophils and neutrophils, can induce immunopathogenic side effects. In RSV-infected mice, NK cells accumulated in the lungs during the early stage of infection and produced large amounts of IFN-γ (Figure 3) [150]. The depletion of NK cells reduced the disease severity and the influx of inflammatory cells like T cells [150]. In studies with nude and wild-type BALB/c mice, increased numbers of NK cells in the lung were associated with airway inflammation and hyperresponsiveness [151]. The depletion of NK cells reduced disease severity and decreased the levels of Th2-type cytokines IL-4, IL-5, and IL-13, indicating that NK cells might contribute to a Th2-skewed immune response at later stages [151]. Direct infection of NK cells might be an explanation for these immune responses. *In vitro* studies have shown that RSV infects neonatal and adult NK cells efficiently [152]. This infection of NK cells was further increased by sub-neutralizing antibodies, indicating the uptake of viral particles in an Fc-γ-receptor-dependent manner [152]. Infected NK cells were characterized by an increased expression of IFN-γ and a decrease in perforin secretion, suggesting a shift from the cytotoxic to the proinflammatory phenotype of RSV-infected NK cells [152]. 

The role of NK cells seems to depend on the stage of infection. In mice infected with recombinant RSV expressing IL-18, increased NK cell numbers were detected at early stages that correlated with increased lung inflammation and reduced viral replication [153]. Depleting NK cells during that infection stage reduced the disease severity and increased the viral load. However, the depletion of NK cells at later stages of infection was associated with increased disease severity mediated by the increased influx of CD8^+^ T cells [153]. This demonstrates the biphasic role of NK cells during RSV infections. 

The NK cell response strongly depends on environmental stimuli. At the early stages of infection, the cytotoxic and immune-recruiting activity of NK cells is required to restrict viral spread. However, the cytotoxic activity of NK cells may also affect uninfected cells and thereby contribute to increased lung pathology. At later stages of infection, as immune cells infiltrate the lung, NK activity must shift from a cytotoxic to a regulatory phenotype to limit immune-cell-mediated lung inflammation. An imbalance in this immune response, induced by direct RSV infection or an altered microenvironment, can lead to increased lung damage.

### 2.5. Monocytes

Monocytes originate in the bone marrow from a common myeloid progenitor and belong to the mononuclear phagocyte system [154]. Monocytes are classified into three groups based on surface markers and function [155]. Classical monocytes represent the largest population (~90%) and are CD14^++^CD16^-^CCR2^hi^CX3CR1^low^ with inflammatory and phagocytic activities [155]. Non-classical monocytes with a CD14^+^CD16^+^CCR2^low^CX3CR1^hi^ phenotype have antiviral and patrolling activities [155]. Intermediate monocytes represent a transitional population between classic and non-classic monocytes and have a CD14^+^CD16^+^CX3CR1^hi^CCR2^low^ phenotype. Intermediate monocytes are proinflammatory and show increased activity in antigen presentation [155]. Mature monocytes circulate in the blood and infiltrate the tissue in response to chemoattractants. In the steady state, monocytes can differentiate into macrophages and dendritic cells in the presence of granulocyte-macrophage colony-stimulating factor (GM-CSF) and macrophage colony-stimulating factor (M-CSF) [156,157]. During viral infections, monocytes and monocyte-derived phagocytes, i.e., macrophages and dendritic cells, contribute to viral clearance by the secretion of antiviral mediators and phagocytosis of infected cells [135,136], as shown for HIV-1 and the influenza virus [158,159,160]. 

Monocytes are recruited to the site of infection during the early phase by IL-6, IL-8, CCL2, CCL5, and MIP-1α derived from infected AECs and macrophages [108,161,162]. The kind of recruited monocytes depends strongly on the secreted cytokines. CCL2 mediates classical inflammatory cytokine recruitment while non-classical, anti-inflammatory monocytes are recruited by CX3CL1 [156]. Alterations in the secretion pattern, e.g., mediated by RSV, could lead to a modified and shifted immune response. Following recruitment and activation, monocytes produce CCL2 and MIP-1α/β, resulting in the recruitment of immune cells [108]. They directly contribute to viral clearance by phagocytosis of the virus in a surfactant A-enhanced manner [163]. In response to uptake, monocytes expressed type I IFNs, TNF-α, G-CSF, and IL-6 [109]. A byproduct of monocyte-mediated viral clearance is the decrease in CCL5 expression by AECs and, consequently, the limitation of immune cell infiltration and cell-mediated immunopathology [109]. Growth factors like G-CSF, however, are secreted continuously and mediate the survival and differentiation of recruited cells [109]. Direct interaction of the RSV F protein with TLR4 and CD14 on the surface of monocytes further initiated innate immune pathways that resulted in the secretion of IL-6, IL-8, TNF-α, and IL-1β, which in turn mediated the antiviral immune response [164]. In TLR4-deficient mice, recruitment of CD14^+^ and NK cells was diminished upon infection while viral clearance was delayed [149,164].

The upregulation of co-stimulatory factors like CD40, CD80, and MHC-I/II on the surface of activated monocytes reflects their role in antigen presentation (Figure 2) [109,165]. Expression of HLA-DR on the surface of monocytes isolated from RSV-infected infants was reduced and correlated with increased disease severity, underlining their importance in antigen presentation and the initiation of adaptive immunity [165]. A previous study analyzed the immune responses in a CD14-deficient patient with recurrent RSV infections and discovered that this deficiency impaired the innate immune response, i.e., a decrease in IL-6 expression, mediated by monocytes to RSV, resulting in inadequate control of infection characterized by recurrent and severe RSV infections [166]. Monocytes, therefore, contribute to viral clearance by phagocytosis of viral particles and by supporting the proliferation, activation, and survival of other immune cells through the secretion of cytokines and upregulation of surface markers. 

Several studies also described the involvement of monocytes in immunopathogenesis. *In vitro* restimulation of monocytes isolated from RSV-infected patients with bronchiolitis (*ex vivo*) showed an increase in IL-10, associated with recurrent wheezing, and decreased levels of IFN-γ, IL-12, and IL-4, indicating a direct contribution of monocytes to immunosuppression during severe infections (Figure 3) [167]. RSV can productively infect monocytes the same as other immune cells [168,169]. The permissiveness of monocytes to infection depended on their maturation and activation state, as *in vitro* studies of isolated cord blood monocytes were more permissive to infection than adult monocytes [168]. Infection of neonatal monocytes induced the expression and secretion of proinflammatory cytokine IL-1β [170]. The infection did not affect their cell viability, indicating that RSV inhibits the apoptosis of monocytes and instead alters their cytokine response [170]. 

The interaction of soluble G with CX3CR1 on the surface of monocytes may lead to altered gene and cytokine expression by inhibiting the nuclear translocation of NF-κB [171]. This change in cytokine response, notably the increased expression of IL-10, may skew for a Th2-typed immune response, a hallmark of severe infections [171]. Following their recruitment to the lungs, monocytes are exposed to different stimuli leading to their differentiation into macrophages with a proinflammatory (M1) or anti-inflammatory phenotype (M2) (described in Section 2.6). Based on this, it can be speculated that RSV alters the lung microenvironment resulting in increased recruitment of non-classical, anti-inflammatory monocytes. Microenvironmental stimuli and possibly the interaction of soluble G with CX3CR1 on their surface may result in alternatively activated, anti-inflammatory monocytes with an M2-like phenotype. This shift in the early immune responses would impair viral clearance. 

Recruiting functional monocytes to the lungs in response to stimuli is pivotal for protection and immune regulation. In addition, monocyte recruitment is crucial to replenish the macrophage population during infection. Despite their protective effects, microenvironmental changes, especially due to G protein interactions, can affect their immune response. A modified cytokine expression following infection in the lung may shift monocyte differentiation toward an anti-inflammatory, M2-like phenotype, resulting in delayed viral clearance.

### 2.6. Macrophages

Macrophages belong to the mononuclear phagocyte system, like monocytes and dendritic cells, and are characterized by their phagocytic activity toward cell debris in different situations, like tissue repair or infections [154,172]. Macrophages circulate in the bloodstream but can migrate and reside in various tissues, like the lung, liver, and brain, in response to microenvironmental stimuli [172]. Two subsets of macrophages are present in the lung: alveolar macrophages, which are present on the surface of the alveolar cavity, and interstitial macrophages, which remain in the interstitium [173,174]. Lung-tissue-resident, alveolar macrophages show two distinct polarization states, M1 and M2, with different functions [156,173]. M1-like macrophages display a proinflammatory phenotype due to the secretion of proinflammatory cytokines, decreased expression of CX3CR1, and the increased expression of CD86, a co-stimulatory surface marker involved in activating T cells, demonstrating their role in antigen presentation [156,173]. M2-like macrophages, on the other hand, are characterized by the increased expression of CX3CR1, and mediate anti-inflammatory responses and contribute to tissue repair [156,173]. As mentioned above, monocytes are recruited to the lungs during viral infection to replenish the macrophage population. It should be noted that monocyte-derived macrophages are a distinct population formed during infection and do not have the role and function of tissue-resident, long-lived macrophages [156]. During the early stages of infection, monocytes differentiate into M1-like macrophages to mediate viral clearance and initiate adaptive immunity [156]. At later stages, monocyte differentiation switches from M1 to M2 polarization to limit the inflammatory response and promote tissue repair [156]. However, macrophage polarization is not irreversible and can shift between the different M-like subsets depending on endogenous stimuli [156]. 

Macrophages can detect RSV through MAVS-coupled RLRs and IFNAR signaling, producing type I IFNs, like IFN-α/β, IL-6, MIP-1α, and CXCL10 (Figure 2) [161,175,176]. It was shown that the macrophage-mediated release of IFNs is crucial for controlling RSV infection as they recruit immune cells to the infection site [161]. Several studies emphasized the role of macrophages in controlling and clearing viral infection. Depletion or impaired activity of macrophages led to increased viral replication, disease severity, inflammation, and excessive neutrophil/dendritic cell infiltration [177]. It is thought that the depletion of alveolar macrophages resulted in a proinflammatory environment characterized by increased expression of G-CSF, IL-17, TGF-β, MIP-1α, and IL-α/β, and decreased levels of TNF-α, IL-6, and type I IFNs [177]. It could thus be speculated that the functional immaturity of neonatal alveolar macrophages leads to an inefficient uptake of infected cells, recruitment of immune cells, and clearance of damaged cells [178]. The activity of different subsets of macrophages appears to be dependent on the stage of infection, similar to the activation pattern of NK cells. During the early stage of infection, M1-like, proinflammatory macrophages are essential for the restriction of viral spread, removal of infected cells, expression of type I IFNs for immune cell recruitment, and antigen presentation to initiate adaptive immune responses. Later during an infection, an M1 to M2 phenotypic switch can be observed, where M2 macrophages produce type III IFNs in a PPAR-γ-dependent manner and secrete IL-10 to control the immune-cell-mediated inflammatory response and mediate tissue repair [179]. M2 differentiation is thought to be mediated by TLR4 and IFN-β signaling [180]. This phenotypic switch is crucial for suppressing exaggerated, unspecific antiviral responses and preventing unnecessary tissue damage. 

A possible contribution of macrophages to immunopathology remains inconclusive and requires further research. Several *in vitro* and *in vivo* studies show the infection of macrophages by RSV [181,182,183]. This infection is TLR4- and CX3CR1-dependent and polarizes the differentiation of neonatal macrophages toward an M2-like phenotype (Figure 3) [180,184,185]. It is unclear whether it is an abortive or productive infection, as various studies yielded different conclusions. While one study shows no loss of cell viability upon infection [181], another indicated that RSV induces necroptosis and suggests an enhancement of viral replication by M2-like macrophages, thus contributing to disease severity and lung pathology [183]. Regardless of that, *in vitro* infection of macrophages, isolated from adult or neonatal humans, has been shown to alter the immune response, characterized by the expression of proinflammatory cytokines like TNF-α, IL-6, and IL-8 [186,187], while the expression of IFN-γ and IL-12, a regulatory cytokine, was impaired [187]. Reduced expression of IFN-γ was associated with impaired activation of macrophages resulting in reduced phagocytosis of the virus and recruitment of immune cells like T cells [188]. These data suggest that the direct interaction with RSV impairs the activation and function of neonatal macrophages by skewing their differentiation toward an M2-like phenotype, thereby contributing to disease severity. 

Macrophage responses during RSV infections follow a biphasic course similar to NK cell response. During the early stage of infection, proinflammatory M1-like macrophages are activated to restrict viral spread. At later stages, probably by the arrival of T cells, a phenotype switch towards an anti-inflammatory, immune regulatory M2-like type is necessary to reduce immunopathogenesis. It can be speculated that in infants with severe infections, the change in the macrophage response is impaired, leading to exaggerated T-cell responses and increased lung damage.

### 2.7. Dendritic Cells

Dendritic cells complete the mononuclear phagocyte system and are considered professional antigen-presenting cells as they bridge innate and adaptive immune responses during infections [189]. Compared to the other two members of the phagocyte system, DCs only display a low phagocytic activity [190]. Two DC subsets have been described based on the expression of surface markers, function, and origin. Conventional or myeloid DCs (cDCs) are involved in tissue damage sensing and the capture and presentation of antigens [191,192]. cDCs are further divided into cDC1s, located directly underneath the airway epithelium and responsible for MHC-I-mediated antigen presentation to CD8^+^ T cells, and cDC2s, located in the lung parenchyma and presenting antigen to CD4^+^ T cells [191,192]. Plasmacytoid DCs (pDCs) are found in blood and lymphoid tissues and are an essential source of IFN-α/β [191,192]. While DCs show reduced phagocytic activity and low antigen presentation under homeostatic conditions, their T cell-priming activity increases during viral infections as co-stimulatory markers, like CD40, CD80, and CD86, are upregulated on the surface [191]. 

Upon infection with RSV, DCs are recruited to the site of infection and sense the virus either through direct infection or by binding viral antigens through PRRs, like TLRs or RLRs [193,194]. As a result, co-stimulatory molecules CD40, CD80, CD83, CD86, and MHC-I/II were found to be upregulated on the surface of infected DCs [195], indicating their maturation (Figure 2). *Ex vivo* studies implied a ‘two-step process’ for maximal maturation of DCs since only the direct infection of pDCs led to increased expression of CD40 [196]. Once DCs are activated and matured, TLR7 and myeloid differentiation factor 88 (MyD88) mediate the production of antiviral and Th1-type cytokines (CCL5, IL-12, IFN-α, and IFN-β) [176,193,197,198]. Matured DCs then migrate to lung-draining lymph nodes to initiate the virus-specific T-cell response and have an essential role in initiating the virus-specific adaptive immune response and, consequently, in controlling RSV infection. 

Despite their protective and crucial role in immunity, DC functions are heavily affected by RSV-induced immune modulatory mechanisms. Several studies showed that the direct infection of DCs impacts subsequent DC-mediated T-cell activation (Figure 3) [199,200]. Upon infection, the RSV N protein is expressed on the surface of infected DCs, which is thought to interfere with DC–T cell synapse formation [201]. This interference impairs or delays the formation of RSV-specific T-cell immunity [201]. Others found a decreased expression of co-stimulatory molecules on infected neonatal DCs that impaired their recruitment to the lymph nodes, ultimately reducing virus-specific T-cell responses compared to the control group [202,203]. The increased expression of IL-4R-α on the surface of neonatal cDC2s, in comparison to cDCs derived from adult mice, may also account for the altered DC responses and impaired recruitment of the cells into the lymph nodes as the deletion of the receptor resulted in increased recruitment to the lymph nodes, reduced Th2 polarization, and upregulation of IL-12 expression [204]. Interaction of the RSV G protein with DC-/L-SIGN inhibits the activation of DCs and interferes with the DC-induced production of cytokines, illustrating an immunomodulatory mechanism by RSV for interfering with the host’s immune response [205]. 

DCs, as the bridge between innate and adaptive immunity, are of major importance for the induction of virus-specific immune responses. The direct infection of DCs and the associated functional changes may lead to severe consequences in initiating a functional adaptive immune response. 

## 3. Adaptive Immune Responses to RSV Infection 

The adaptive immune response represents the second line of defense against infections. Although innate immunity reacts to infection rapidly, the recognition of pathogen patterns is limited since the responsible receptors are germline-encoded [206]. Adaptive immune responses, on the other hand, are characterized by highly specific receptors on the surface of the lymphocytes allowing targeted, pathogen-specific responses. Activation of adaptive immune cells leads to their proliferation and clonal expansion, exerting their effector function, and ultimately to the generation of an ‘immunological memory’ that persists in the host and can be rapidly reactivated during reinfection [207,208]. The adaptive immune system comprises two cell types: T lymphocytes and antibody-secreting B cells [207]. Mature adaptive immune cells reside in secondary lymph nodes and can infiltrate the site of infection after stimulation with antigens presented by innate immune cells, like monocytes, macrophages, or DCs [207]. In the following part, the protective roles of B and T cells and the potential impairment of their function by RSV during infections will be discussed in more detail. 

### 3.1. B Cells

B cells originate in the bone marrow from hemopoietic stem cells [209]. In an immature state, immunoglobulin (Ig)-expressing B cells leave the bone marrow and migrate to secondary lymphoid organs for their final development and maturation [209]. Final B cell activation is mediated by two distinct processes: T-cell-independent and -dependent activation, both of which require the binding of an antigen to the B cell receptor (BCR) [209]. During T-cell-independent activation, antigens with multiple, repeating epitopes are necessary for the cross-linking of BCRs, leading to the proliferation and differentiation of B cells into antibody-secreting plasma cells [209]. For the majority of antigens, however, B cell activation requires the help of CD4^+^ T helper (Th) cells. During this process, B cells internalize the antigen and present it on the cell surface through MHC-II. The antigen is then recognized by antigen-specific Th cells that initiate B cell proliferation and differentiation by transmitting activating signals [209]. Besides the generation of antibody-secreting plasma cells, the T cell-dependent activation of B cells also allows the development of B memory cells. B cell development is completed by somatic hypermutation, affinity maturation, and isotype switching, all of which result in the production of highly specific antibodies [209]. Upon viral infections, B cells secrete virus-specific antibodies, neutralizing the virus and preventing viral cell entrance by blocking the viral binding domain [210]. B cells and antibodies, therefore, are an important correlate of protection by eliciting virus-specific effector functions. 

Following RSV infection in infants and adults, B cell numbers in the peripheral blood were increased compared to uninfected individuals. They displayed a differentiated phenotype by the expression of typical proliferation, differentiation, and survival markers, such as B cell-activating factor (BAFF) and A proliferation-inducing ligand (APRIL) (Figure 2) [211,212,213,214,215]. These observations are supported by studies in mice where RSV infection led to the upregulation of BAFF, APRIL, and CXCL13 on B cells [216,217]. These studies demonstrate the activation and circulation of B cells following RSV infection and indicate their migration to the site of infection. Although B cells are activated and recruited during RSV infection, studies suggest that these numbers rapidly decrease once the infection is cleared [218,219,220]. In blood samples of infected adults, low levels of RSV-specific B cells were detected in healthy ‘non-healthcare’ workers [219]. After experimental infection of the volunteers, the numbers of RSV-specific B cells rapidly decreased within months after exposure [219]. *Ex vivo* studies confirmed these observations after the experimental infection of adults in which RSV-specific B-cell responses generated in the volunteers were poorly maintained and returned to baseline levels one year after exposure, indicating a defective memory B-cell response in adults to a certain degree compared to samples from RSV-negative, hospitalized infants [220]. 

RSV-specific, neutralizing antibodies can be detected in humans as early as two days after infection [213]. They mainly target the RSV glyco- and fusion protein (preF and postF confirmation) and display a cross-reactivity for both RSV subtypes [212,221]. RSV-specific antibodies can mediate viral clearance through various mechanisms, like ADCC, antibody-dependent cellular phagocytosis, and the neutralization of viral particles [221]. Their protective role was confirmed in mice, where administering RSV-specific, neutralizing antibodies before RSV exposure reduced the morbidity and mortality of the animals compared to IgG-treated controls [222]. The presence of these neutralizing antibodies before the RSV challenge further restricted virus replication in the early stage of infection and thereby reduced the activation of CD8^+^ memory T cells that displayed immunopathogenic activity in control mice through the increased secretion of IFN-γ [222]. In earlier studies with older adults, low RSV-neutralizing serum and mucosal antibody levels prior to infection correlated with disease severity [21,223,224,225,226,227]. Upon RSV infection, older adults develop stronger nasal and serum antibody responses than younger subjects, which is dependent on the duration of viral shedding [213,228]. In a recent study, older adults mounted poor nasal IgA responses, but robust serum IgG responses that correlated with recovery from the RSV infection [226]. Levels of RSV-specific, neutralizing antibodies, however, were relatively short-lived and titers dropped more than 4-fold within a year after infection in the majority of study subjects [218]. Despite the fact that older adults have comparable antibody levels to younger subjects prior to infection, they are more susceptible to severe RSV infections, which indicates that other immunological parameters, like dysfunctional T cells, may account for this [229,230]. 

Although antibodies mediate viral clearance, they may also contribute to immunopathogenesis during RSV infections. Isolated RSV-specific antibodies and B cells of infected infants show significant differences from those of adults [231]. F-specific antibodies of infants under 3 months of age showed restricted affinity, neutralization capacity, and recognition of antigenic sites compared to adult samples [231,232]. The RSV F protein contains six major antigenic sites (ø, I–V), of which only antigenic sites I and III were recognized by antibodies isolated from infants. Antibodies directed against antigenic site I do not display neutralizing activity and their preferred production in infants could result in lower levels of neutralizing antibodies. A high abundance of non-neutralizing antibodies in infants could ultimately result in the formation of immune complexes that may contribute to lung pathology if not cleared in time [231]. The defective generation of neutralizing antibodies seems to be age-dependent in childhood as the recognition of additional epitopes increases with age [231]. A genetic limitation in infants under three months of age may be responsible for this defect as infant B cells have limited use of antibody variable genes and lack somatic hypermutation [233]. Maternally derived antibodies may provide an explanation for this limitation since several studies indicated a suppressive effect of maternal antibodies on the infant’s immune response in the first months of life during RSV infection *in vivo* [219,234,235]. Recent immunization experiments in infant mice indicate that maternal antibodies altered B cell differentiation and isotype switching, thereby limiting the B cell repertoire [236].

The virus itself further affects B-cell responses. Analysis of neonatal regulatory B cells (nBregs) isolated from cord blood demonstrated their infectability by RSV [237]. The interaction of the RSV F protein with the B cell receptor activated the regulatory B cells and increased the expression of CX3CR1, a potential viral receptor, via the CX3C motif of the RSV G protein. Therefore, the interaction of RSV F with BCRs on the surface of nBregs leads to increased expression of CX3CR1 on the surface of the cells, making them susceptible to direct infection. The infection of nBregs resulted in the secretion of IL-10, an immunoregulatory cytokine, which reduced the production of Th1-type cytokines [237]. This study, therefore, demonstrates the immune modulatory effects mediated by RSV F and G on nBregs, ultimately resulting in an immune response that favors viral survival (Figure 3) [237]. Besides the direct infection of nBregs, the secretion of soluble G may depict another immune modulatory effect on B cell activity. Soluble G acts as an antibody decoy during infections, thereby serving as an escape mechanism for viral progeny from G-specific antibodies and interfering with antibody-mediated viral clearance [238].

Antibodies are the major correlate of protection during RSV infection in infants, as shown by the prophylactic use of human polyclonal neutralizing antibodies [239,240,241] and the neutralizing monoclonal antibodies palivizumab and nirsevimab [242,243]. The presence of pre-existing, RSV-specific antibodies and, therefore, memory B cells during reinfections is crucial, as they are able to detect the virus before the infection of immune cells. Memory B cells thus have a relatively long-lasting effector function, while the effector functions of memory T cells are limited to the presence of antigen [244]. 

Direct modulation by RSV G and the genetic limitations of the immune system in infants under 3 months of age interfere with the generation of neutralizing antibodies, leaving the infant at risk of more severe infections. Direct infection of nBregs resulting in the secretion of immunoregulatory IL-10 demonstrates that interactions of viral proteins can shift the immune response, generating an environment that favors viral shedding. Although RSV-specific immune responses have been studied extensively in infants, the induction of these responses in older adults has been underinvestigated and warrants more research, especially in the context of age-related immunosenescence [245].

### 3.2. T Cells

Unlike B cells, T cells arise from a common lymphoid progenitor in the thymus [208]. T cells migrate to secondary lymphoid tissues upon maturation to encounter antigens [207,208]. Two major subsets of T cells have been described, characterized by the expression of either CD4 or CD8 on their surface. Both subsets are activated by the formation of a so-called ‘immunological synapse’ which describes the interaction of different T cell markers, e.g., CD4/CD8, CD3, or T cell receptor, with antigen-bearing MHCs on the surface of professional (MHC-II; DCs, macrophages, monocytes, and B cells) and non-professional (MHC-I; epithelial cells) antigen-presenting cells [207,246]. Activated T cells can exert various effector functions, such as the elimination of infected cells, activation of other immune cells, like B cells, through the secretion of cytokines or direct interaction, or regulation of immune responses to prevent excessive damage [247]. In the following part, the protective role of the individual T cell subsets as well as their contribution to immune pathogenesis during an RSV infection will be illustrated in more detail.

#### 3.2.1. CD4^+^ T Cells

CD4^+^ T cells are pivotal in the host’s immune response as they orchestrate the activity of innate and adaptive immune cells through the secretion of several cytokines. They can be divided into different subsets (i.e., Th1, Th2, Th9, Th17, T-follicular helper, and Tregs), which mainly provide helper functions and are characterized by the secretion of specific cytokines [247]. For example, Th1-like cells are involved in activating mononuclear phagocytes or cytotoxic T cells by the secretion of IFN-γ, TNF-α, IL-2, IL-12, and IL-18. Th2-like cells, by contrast, are involved in eosinophil responses, and B cells maturation, and production of virus-specific antibodies by secreting IL-4, IL-5, IL-10, and IL-13 upon activation [247]. 

During RSV infection, CD4^+^ T cells are recruited to the airways and orchestrate the immune response via secretion of IL-17, IFN-γ, and TNF-α (Figure 2). The adoptive transfer of CD4^+^ T cells into nude mice demonstrated their beneficial role during infection and B cell differentiation as they were necessary to induce the production of RSV-specific antibodies [248]. Adoptive transfer of CD4^+^ T cells from the airways of RSV-infected mice into naïve mice reduced disease severity by suppressing the secretion of TNF-α compared to control mice, further supporting their protective role during infection [249]. In human experimental infection studies, numbers of RSV F- and G-specific, CD4^+^ T cells were increased in the airways of infected individuals [250]. By contrast, in infants with severe RSV infection with subsequent wheezing, numbers of CD4^+^ T cells were reduced and produced lower levels of TNF-α after *in vitro* restimulation compared to infants without wheezing, indicating a partial impairment of the CD4^+^ T-cell responses in these children [148,251,252]. The production of proinflammatory cytokines by CD4^+^ T cells during viral infections is crucial for the activation and regulation of innate and adaptive immune responses. However, the balance between pro- and anti-inflammatory cytokine responses is of great importance, and any alteration in this balance may have serious consequences, as illustrated by the use of TNF-α. The studies mentioned above show that an imbalanced TNF-α response during RSV infections can lead to more severe disease and prolonged viral shedding. 

A dysfunctional CD4^+^ T-cell response during infection was linked to immunopathology after FI-RSV vaccination [253,254]. Studies in mice vaccinated with FI-RSV showed a Th2-skewed immune response characterized by the increased expression of Th2-type cytokines like IL-5 and IL-13, resulting in airway hyperresponsiveness (AHR) and increased mucus secretion compared to mock-immunized controls [255]. Depletion of CD4^+^ T cells in the mice significantly improved the condition of the mice during infection, indicating that a biased Th2-type immune response contributes to disease severity in FI-RSV-vaccinated mice [255]. Similarly, RSV infection of neonatal mice and their reinfection in the adult phase led to an exaggerated Th2-type immune response resulting in AHR, mucus hypersecretion, and eosinophilia compared to mice that were infected during adulthood [256]. The increased expression of IL4-Rα on the CD4^+^ Th cells of these mice indicates a preferential proliferation of CD4^+^ T cells to a Th2-type [256]. In experimental infection studies of adults, increased levels of CD4^+^ T-cell-derived IFN-γ, IL-2, IL-4, IL-10, and TNF-α and the number of CD8^+^IFN-γ^+^ T cells correlated with increased disease severity and hospitalization, emphasizing the potential immunopathogenic role of CD4^+^ T cells during RSV infection [257]. Depletion of CD4^+^ T cells, IL-10, or IL-4 before infection or vaccination ameliorated disease severity but consequently resulted in prolonged viral shedding [118,255,258,259,260]. It has been shown that RSV can productively infect CD4^+^ and CD8^+^ T cells, resulting in the decreased expression of IFN-γ and interfering with the T cell functions and development of Th1 cells, probably in an F-protein-dependent manner (Figure 3) [261,262]. This direct infection may hinder the generation of long-term protective immunity since RSV-specific lymphocyte responses only persist for about a year and do not boost virus-specific cell-mediated immunity upon reinfection [263]. 

A balanced CD4^+^ T-cell response is pivotal for developing protective immune responses by coordinating the recruitment and activity of innate and adaptive immune cells through the secretion of cytokines. Direct infection by RSV and partial genetic dysfunction of the infant’s CD4^+^ T-cell response, however, may interfere with this balance, leading to a Th2-skewed immune response and more severe infections.

#### 3.2.2. CD8^+^ T Cells

CD8^+^ T cells can recognize and eliminate virus-infected cells through their cytotoxic activity. During RSV infection, naïve CD8^+^ T cells are activated through the interaction of the T cell receptor either with MHC-I on the surface of infected epithelial cells or with MHC-I on the surface of APCs in lung-draining lymph nodes (cross-presentation) [195]. Following activation, virus-specific effector CD8^+^ T cells expand and migrate to the site of infection in response to chemoattractants CCL5 and CXCL10 [264,265]. Activated CTLs directly interact with antigen-bearing MHC-I on the surface of RSV-infected cells and induce the apoptosis of the target cell by the secretion of perforin and granzymes [266,267,268]. 

Several studies revealed a protective role of CD8^+^ T cells during RSV infection. The depletion of CD8^+^ T cells in RSV-infected mice resulted in delayed viral clearance compared to control mice. The adoptive transfer of CTLs, isolated from RSV-infected mice, into naïve mice during RSV infection reduced both the viral load and weight loss [249]. These results are supported by human studies analyzing the blood and tissue samples of infected infants, in whom reduced CD8^+^ T cell counts were associated with delayed viral clearance and increased disease severity in comparison to uninfected infant samples, emphasizing their protective role during infection [148,252,269,270]. In an RSV-infected infant with severe immunodeficiency, bone marrow transplantation significantly reduced the nasal viral load [271]. This decrease in viral load began with the increasing activity of CTLs after transplantation [271]. CTLs mediate their effector functions during RSV infection by the secretion of several cytokines, e.g., IL-2, IL-10, INF-γ, TNF-α, and the serine protease granzyme B (Figure 2) [264,266,267,272,273,274,275]. The importance of CD8^+^ T-cell-derived cytokines is demonstrated by studies in mice that were primed for an RSV-specific CD8^+^ T-cell response prior to RSV challenge [276]. In the primed mice, CD8^+^ T cells suppressed the exaggerated Th2-type cytokine response characterized by the decrease in IL-4 and IL-5, and consequently led to the suppression of excessive recruitment of eosinophils compared to controls. Thus, it can be assumed that CD8^+^ T-cell-derived cytokines contribute to the regulation of CD4^+^ T-cell responses [276]. 

Despite their crucial role in viral clearance, CTLs are also involved in immunopathology following RSV infection. RSV-infected mice displayed increased levels of CTL-secreted IFN-γ that correlated with disease severity in comparison to control mice [277]. Depletion of CTLs in mice markedly reduced disease severity following RSV infection, although viral shedding was prolonged, indicating that CTLs are involved in both protective and harmful immunity [258]. In BAL samples of hospitalized, RSV-infected infants, increased levels of granzymes A and B were detected compared to children without a pulmonary disease (Figure 3) [278,279]. The increased levels of granzymes correlated with IL-8 levels in the airways and disease severity [278,279]. In experimental infections of adults, the peak of CTL numbers in the respiratory mucosa was linked to the reduction in viral load and an increase in symptoms, emphasizing the dual role of CTLs during RSV infection [266]. Several studies have implicated CTL-derived IFN-γ and TNF-α as a driving force in CTL-mediated immunopathogenesis during RSV infection. Increased levels of IFN-γ in the BAL of RSV-infected mice correlated with disease severity, while its depletion with neutralizing antibodies or absence in IFN-γ-KO mice led to ameliorated disease [277,280]. A similar role in immunopathogenesis was shown for TNF-α. Its neutralization in mice before RSV infection reduced weight loss [281]. 

These data emphasize the role of CTLs in both virus elimination and immunopathogenesis. While CTLs mediate viral clearance through their cytotoxic activity and are involved in the regulation of a balanced CD4^+^ T-cell response, their excessive activation and infiltration of the lung tissue may contribute to lung pathology and disease severity characterized by the increased secretion of granzymes.

#### 3.2.3. Regulatory T Cells

Previously discussed immune cells, such as B and T cells, illustrate the importance of a balanced immune response during viral infection, like SARS-CoV-2 or hepatitis B virus [282,283]. Tregs are a subset of CD4^+^ T cells characterized by the expression of CD25 and the transcription factor forkhead box protein 3 (FoxP3), which is pivotal for their development and regulatory function [284]. Tregs can control immune cell recruitment and proliferation and prevent exaggerated immune responses during infections by secreting TGF-β and IL-10 [285,286,287,288]. 

During RSV infection, Tregs accumulate early in the lungs and lymph nodes of mice [275,289,290]. The depletion of Tregs in mice resulted in the delayed recruitment of CTLs and CD4^+^ T cells to the site of infection, followed by delayed viral clearance, increased weight loss, and slower recovery compared to naïve control mice, indicating that Tregs mediate the recruitment of T lymphocytes during RSV infection [117,290,291,292,293]. Despite the delayed recruitment of CTLs and CD4^+^ T cells after Treg depletion in mice, their numbers were significantly increased later. They were associated with an excessive inflammatory response later during infection, characterized by the increased secretion of IFN-γ and TNF-α (Figure 2) [117,290]. Exaggerated and persistent Th2-like responses, characterized by IL-13-expressing CD4^+^ T cells, were also noticed in the absence of Tregs, further illustrating the role of Tregs in the regulation and suppression of immunopathology during RSV infection [117]. *In vivo* studies showed an increased expression of granzyme B by lung-resident T cells compared to naïve mice, suggesting that the expression of granzyme B mediates the regulatory activity of Tregs and that Treg-derived granzyme B may eliminate activated lymphocytes, thereby regulating inflammatory responses [292]. Since Tregs are a major source of IL-10, their immunosuppressive function might be mediated through an IL-10-dependent mechanism. Studies in IL-10^−/−^ mice show an increased accumulation of CTLs, increased levels of proinflammatory cytokines, like IFN-γ and TNF-α, and increased disease severity in the lungs of RSV-infected mice in comparison to control mice [294,295]. The absence of Tregs in mice further indirectly affected immunity to RSV as decreased concentrations of neutralizing and increased concentrations of non-neutralizing antibodies were measured compared to those in naïve mice [293]. These data underline the versatile role of Tregs during RSV infection. 

In infants with severe infections, the numbers of activated Tregs were reduced in blood and nasal aspirates compared to uninfected children and remained at that level for several weeks after infection [296,297]. The decrease in IL-33 levels, a cytokine involved in the accumulation and homeostasis of Tregs in mucosal sites, in the nasal aspirates of RSV-infected infants, may explain the reduced numbers of Tregs in these samples (Figure 3) [296]. The reduced accumulation of Tregs and lower levels of IL-33 in these infants may be the consequence of a Th2-type cytokine milieu. The Th2-type environment, characterized by the increased secretion of IL-5 and IL-13, induced the Th2-like development of the Tregs effector phenotype and a loss of its immunosuppressive function [298]. Th2-like Tregs were characterized by the expression of GATA-3, a transcription factor that can inhibit the expression of FoxP3 in Tregs [298]. Since Tregs are involved in cell recruitment, especially during the early stage of infection, a decrease in their activity would mean a delayed infiltration of immune cells into the tissue, e.g., CTLs, and, consequently, a delay in viral clearance [290]. 

Tregs and their activity are crucial to limiting immunopathology during RSV infection, probably mediated by granzyme B and IL-10. Alteration in the host immune response to RSV infection towards a Th2-like phenotype indirectly interferes with Treg activity, resulting in delayed and increased recruitment of immune cells and exaggerated responses.

#### 3.2.4. Memory T Cells

Memory T cells are essential for the rapid, antigen-specific immune response during virus reinfection. Following viral clearance, the virus-specific T cell population contracts, leaving only a small fraction of cells to survive, which differentiate into persisting memory T cells [299]. Memory T cells can be classified into four subsets based on surface marker expression [300]. Following infection, central memory T cells (CD45RA^-^CCR7^+^) home in on secondary lymphoid organs, while effector memory T cells (CD45RA^-^CCR7^−^) migrate into the peripheral tissue to mediate effector functions [300]. Late effector memory T cells are considered terminally differentiated and have a reduced functional capacity [300]. Tissue-resident memory T cells (Trm), characterized by the increased expression of CD69 and CD103, represent the last subset and reside within the peripheral tissue [266,301]. Lung Trms are further characterized by the expression of CD11a and CD49a [302].

During secondary infections with RSV, memory cell populations rapidly expand and induce a virus-specific immune response in a MAVS- and IFN-α-dependent manner [195,303,304]. Memory T cells in mice are recruited and activated in three waves: the initial activation of tissue-resident memory T cells in the lungs is followed by the secondary recruitment of circulating, RSV-specific memory cells from the periphery. The third wave consists of memory T cells activated by antigen-presenting cells in the lung-draining lymph nodes [305]. The fast response mediated by memory T cells leads to early virus recognition and elimination, reducing the disease severity and viral load [266]. In blood samples of young and elderly volunteers, resting CD8^+^ memory T cells were identified that were characterized by an increased expression of CD27, CD28, and IL-7Rα (Figure 2) [303]. *In vitro* restimulation of these memory cells led to the production of IFN-γ and granzyme B, demonstrating that they can regain their effector functions and proliferate and expand during RSV infection [303]. Similar results were shown in RSV-infected mice [306]. The adoptive transfer of tissue-resident memory T cells into naïve mice before infection reduced weight loss and the viral load compared to control mice showing the protective effect of Trms during RSV infection [249]. 

Because repeated RSV infections are common, an impairment of RSV-specific memory T cell generation may be assumed (Figure 3). In mice, RSV infection induces RSV-specific Trms that correlate with viral clearance and reduced disease severity [306]. However, the number of RSV-specific Trms rapidly waned within five months, corresponding to the age of a young adult in humans [306]. Prime and challenge studies in mice showed similar results as RSV-specific Trms displayed an impaired IFN-γ response, and the numbers of Trms declined rapidly after the clearance of infection [307]. This defect in RSV-specific Trm response was restricted to the respiratory tract, indicating an immunosuppressive effect of RSV on the effector functions and the generation of memory of local T cells after infection [307]. Preexisting, RSV-specific CD8^+^ memory T cells mediate viral clearance in the absence of RSV-specific CD4^+^ T cells and antibodies in mice, emphasizing the protective role of CD8^+^ memory T cells in the absence of other adaptive immune cells [308]. Besides their protective role, CD8^+^ memory T cells were also involved in excessive weight loss and increased disease severity and immunopathology in infected mice mediated by the increased secretion of IFN-γ compared to control mice [308]. This study demonstrates that memory T cells mediate viral clearance upon reinfection but also lead to increased disease severity and immunopathogenesis in the absence of regulation factors like CD4^+^ T cells and antibodies. In a recent study, peripheral blood mononuclear cells were isolated from healthy children who encountered RSV infection during infancy. Restimulation of these PBMCs showed an impaired memory T-cell response irrespective of the disease severity during RSV infection when compared to children that were not infected during infancy [309]. The altered memory T-cell response was indicated by reduced IFN-γ, TNF-α, and IL-2 secretion. This study demonstrates that RSV infections during infancy can attenuate the memory Th1- and Th17-cell responses to RSV later in life regardless of the disease severity of the primary infection or reinfection [309]. 

The reduced survival of memory T cells may be due to intrinsic cell programming, as studies in infant mice infected with influenza virus showed an increased expression of T-bet and a reduced expression of survival factor CD127 on the surface of T cells compared to infected adult mice. This altered phenotype persisted even after the adoptive transfer of infant Trms into adult mice, indicating that the host environment is not responsible for the inefficient establishment of memory and that infant Trms are intrinsically programmed for short-term immunity [310].

Generating memory T cells is essential for long-term protection against recurrent infections. Fully functional memory T cells are produced during RSV infections that can mediate protection upon reinfection. The rapid decrease in RSV-specific memory T cells and impaired functionality in infants and older adults indicate that this protection is not long-lasting and predisposes the host to recurrent infections. Further studies will be necessary to better understand the role of memory T cells during RSV infection and immunopathogenesis, and the role of RSV in regulating these processes.

## 4. Vaccine Development and Treatment Options with Novel Insights into Immune-Mediated Protection

**Figure 4:**
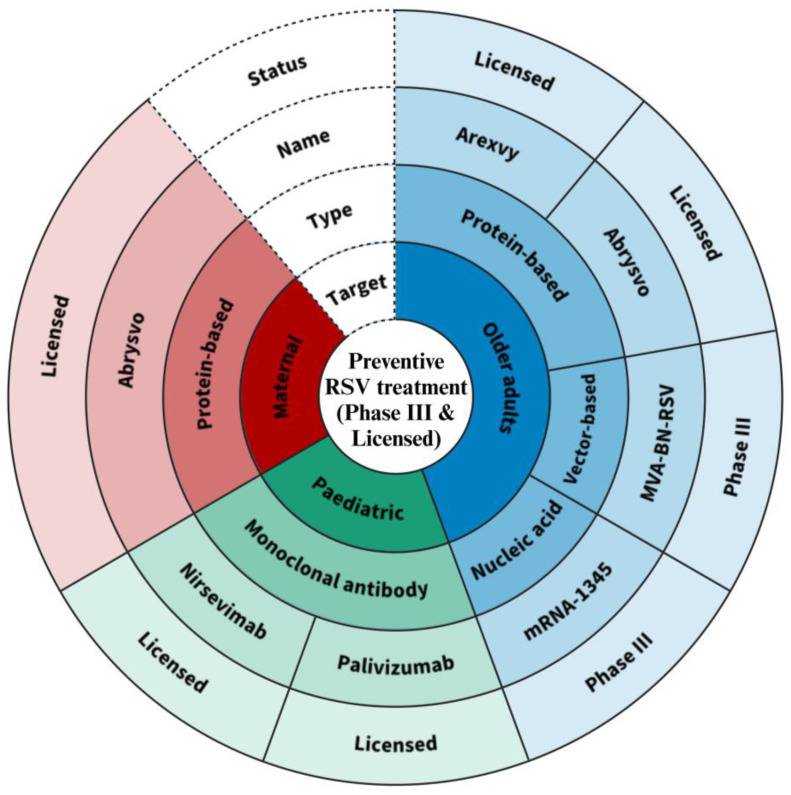
Schematic overview of current vaccines and monoclonal antibody-based treatment options available for RSV. The mentioned vaccines and monoclonal antibodies are either licensed or currently in phase III clinical trials for the treatment of patients at risk. Created with BioRender.com.

After the first isolation of RSV from chimpanzees in 1956 and infants in 1957, the first formalin-inactivated vaccine, FI-RSV, was developed in the 1960s [311,312] and used in a cohort of infants in the USA. Vaccination with FI-RSV led to an increased frequency and severity of ALRIs in seronegative infants following their first natural infection and even led to the death of two vaccine recipients [29]. Possible explanations for ERD in children were discussed above (see Section 2.2, Section 2.3, and Section 3.2.1). For over six decades, the search for an effective vaccine continued without success. RSV vaccine and human monoclonal antibody development changed drastically with the stabilization of the F protein in its prefusion conformation as antibodies directed against prefusion epitopes have a tenfold increase in neutralizing activity compared to antibodies directed to postfusion epitopes [313]. Recently, two stabilized, prefusion F-protein-based vaccines, Arexvy (GSK) and Abrysvo (Pfizer), were licensed for the prevention of ALRI in older adults. Abrysvo was recently approved for the vaccination of pregnant women in their third trimester for the prevention of RSV-induced ALRI in infants during their first RSV season. Since a broad range of RSV vaccines and treatment options are currently in clinical trials, we focus on the ones that are either licensed or are/were in phase III clinical trials (Figure 4). 

Vector-based vaccines have been used for decades for several pathogens, showing their potential to induce protective immunity in recipients after vaccination. They are generated by integrating a viral sequence encoding a target antigen into a non-pathogenic, often replication-deficient viral vector, like modified vaccinia virus or adenoviruses [314]. Vector-based vaccines generally aim to induce a robust antibody and CTL response and are characterized by high immunogenicity. The advantages of vector-based vaccines are the simple and safe production of a high number of vaccine doses [314]. Among the candidates for RSV, a modified vaccinia virus Ankara (MVA)-based vaccine expressing the RSV N, F, G, and M2 proteins (MVA-BN-RSV) is currently in phase III trials for Bavaria Nordic. MVA-BN-RSV would be recommended for use in older adults since vaccination induces stronger T-cell responses than antibody responses. In clinical trials, vaccination induced moderate neutralizing antibody responses and enhanced Th1-type immune responses indicated by the low levels of IL-4 in vaccinated individuals compared to placebo controls. A booster shot after 12 months further increased the antibody titers and T-cell responses in vaccine recipients [315,316]. Phase III clinical trials for MVA-BN-RSV are currently ongoing (NCT05238025). Although vector-based vaccines elicit strong T-cell responses, preexisting immunity to adenoviruses or poxviruses (in smallpox-vaccinated adults) may interfere with vaccine efficacy. Ad26.RSV.preF is an adenovirus-vectored vaccine candidate developed by Janssen Pharmaceuticals which expresses the preF protein. Similar to MVA-BN-RSV, it is designed for use in older adults and is additionally suitable for seropositive children. First trials with Ad26.RSV.preF resulted in increased antibody titers, reduced risk of ALRI, and reduced viral load in comparison to the unvaccinated group [317,318,319]. In March 2023, Janssen Pharmaceuticals discontinued its phase III vaccine development strategy based on Ad26.RSV.preF.

In contrast to whole virus vaccines, subunit vaccines are based on one or more purified viral antigens [320]. Therefore, the choice of antigen is critical in generating protective immune responses. Subunit vaccines are relatively safe as reversion to virulence is not possible, and a spread to unvaccinated individuals can be ruled out [320]. Compared to whole-virus vaccines, however, subunit vaccines must generally be delivered together with an adjuvant, and multiple doses may be necessary because of reduced immunogenicity [320]. A protein-based, bivalent vaccine directed against the prefusion F protein of both RSV subtypes (RSVPreF) was designed by Pfizer for the vaccination of pregnant women and older adults. Intramuscular vaccination of healthy adult men and nonpregnant women (18–49 years) as well as older adults (50–85 years) with RSVPreF induced robust neutralizing antibody responses against both RSV subtypes, with elevated titers through 12 months post-vaccination, while being safe and well tolerated [321,322,323]. In phase III trials of RSVPreF in older adults, vaccination efficiently prevented RSV-associated ALRI in the vaccine recipients compared to the placebo group [324]. Consistent with previous studies, RSVPreF was safe and well-tolerated [324]. In May 2023, RSVPreF, marketed as Abrysvo, was approved by the FDA for the vaccination of older adults (≥60 years) for the prevention of RSV-associated ALRI. Phase III trials for RSVPreF in older adults are currently ongoing to evaluate the efficacy and safety of a second dose of RSVPreF throughout two RSV seasons (NCT05035212). Single-dose vaccination of pregnant women with RSVPreF, administered by the beginning of their third trimester, showed the induction of neutralizing antibodies and efficient transplacental migration of these antibodies to the fetus. Maternal vaccination effectively reduced the risk of ALRI and protected infants from RSV infection during the first six months of life compared to the placebo group [325,326]. The vaccine, marketed as Abrysvo, was recently approved by the FDA for the vaccination of pregnant women during their third trimester representing the first vaccine targeting the infant population. Phase III trials for maternal vaccination are currently ongoing to further evaluate the efficacy and safety of the vaccine in pregnant women and infants (NCT04424316).

A similar subunit vaccine, RSVPreF3, is based on a prefusion F protein antigen and was developed by GSK. Intramuscular vaccination of healthy pregnant women during their late second or third trimester with unadjuvanted RSVPreF3 was safe and induced neutralizing antibodies that were transferred to the fetus [327]. Transplacentally derived antibodies in newborns were high and had waned after approximately six months [327]. In February 2022, GSK stopped recruitment for their phase III trials due to safety concerns, as premature birth was more likely in vaccinated women compared to the placebo group (NCT04605159, NCT04980391, and NCT05229068). Single-dose vaccination of older adults with RSVPreF3 (RSVPreF3 OA) adjuvanted with AS01_E_, administered intramuscularly, significantly reduced the risk of ALRI in vaccine recipients compared to the placebo group [328]. The generated antibodies were detectable for at least six months. Vaccination was safe and displayed a similar efficacy regardless of RSV strain, age group, or comorbidities [328]. In May 2023, RSVPreF3 OA, marketed as Arexvy, was approved by the FDA for the vaccination of older adults (≥60 years) to reduce the risk of ALRI during an RSV season. Phase III clinical trials for RSVPreF OA are currently ongoing to monitor the effects of repeated vaccination and the long-term effects of vaccination in volunteers (NCT04886596). 

During the SARS-CoV-2 pandemic, mRNA vaccines against COVID-19 attracted much interest. These mRNA-based vaccines, coding for a specific viral antigen, are administered intramuscularly using lipid nanoparticles [329]. After translation, mRNAs are degraded, reducing toxicity and the risk of side effects. The successful vaccination campaign during the pandemic also showed that this vaccine system could be safe and immunogenic in different age groups [329]. Although mRNA vaccines can be produced fast and on a large scale, they tend to be relatively unstable and degrade quickly if not stored properly [329]. mRNA-1345, which was developed by Moderna, is a nucleic-acid-based vaccine encoding a membrane-anchored RSV F protein stabilized in the prefusion conformation. Single-dose vaccination of older adults with mRNA-1345 resulted in increased antibody titers compared to the unvaccinated control group, which persisted for six months and effectively prevented RSV-associated ALRI in vaccine recipients [330]. Vaccinations were well-tolerated in older adults without safety concerns. Phase III studies for mRNA-1345 are currently ongoing to further evaluate the efficacy and safety of the vaccine as primary efficacy endpoints in older adults were recently met (NCT05127434). 

Prophylactic treatment with monoclonal antibodies represents an alternative to vaccination. Palivizumab, a humanized IgG1-type monoclonal antibody, was approved in 1998 to treat high-risk infants during their first RSV season [243,331]. Palivizumab targets the antigenic site II of the F protein and prevents viral infection and subsequent replication. Although antigenic site II is present on both preF and postF confirmation, antibodies directed against this epitope display a reduced neutralizing activity compared to antigenic site Ø, which is only present in the preF confirmation [332,333]. Trials in high-risk infants, to whom palivizumab was administered every 30 days for five months, showed reduced RSV-associated hospitalization and disease severity following intramuscular injection while being well tolerated compared to the placebo group [243,331]. Due to palivizumab’s relatively short half-life and the necessity of monthly administrations during RSV season associated with high costs, the American Academy of Pediatrics recommended using palivizumab for high-risk infants during their first RSV season [334], advice that was followed in several other countries [335]. Recently, a new monoclonal antibody, nirsevimab, was approved. This recombinant human IgG1-type monoclonal antibody is directed against the antigenic site Ø in the RSV F prefusion confirmation. A triple amino acid substitution in the Fc domain increased nirsevimab’s half-life, giving it a significant advantage over palivizumab [336]. A single-dose administration of nirsevimab to preterm infants before the RSV season significantly reduced the risk of ALRI and hospitalization compared to placebo-treated infants [337,338]. This effect lasted the whole season and was protective against both RSV serotypes without any safety issues [337,338]. Nirsevimab thus has two critical advantages over palivizumab: the higher neutralization activity of the antibodies and the increased half-life, which means that a single dose before the start of the RSV season is sufficient for the protection of high-risk infants. This makes nirsevimab more cost-effective. The recent approval of nirsevimab offers at least a cost-effective and efficient alternative to vaccination for the infant population. However, it is unclear if RSV can accumulate resistance mutations towards the neutralizing activity of nirsevimab, which has been the case for palivizumab [339]. 

The stabilization of the prefusion F protein is a critical factor for the development of vaccines and human monoclonal antibodies for the prevention of severe RSV infections in infants, older adults, and the immunocompromised. The progress in vaccine development is best illustrated by the recent approval of Arexvy and Abrysvo, which offer protection for the older population from RSV-associated ALRI. For infants, the vaccination of pregnant women seems an attractive option as Pfizer’s PreF-protein vaccine was approved recently by the FDA based on promising data from phase III clinical trials. 

## 5. Concluding Remarks

Despite decades of research, RSV remains a substantial global burden, mainly affecting infants, older adults, and immunocompromised individuals. After the early failure of FI-RSV-based vaccines in the 1960s, research has focused on understanding the host’s immune response and possible immunomodulatory effects of RSV that may counteract a protective response and may even enhance immunopathological effects. This review emphasizes that effective and well-balanced immune responses are fundamental for reducing RSV-associated disease. As summarized, RSV-altered immune cell activity and differentiation robustly impact the cellular and humoral immune response. This dysregulation is mainly characterized by a Th2-skewed immune response leading to more severe disease and immunopathology. In the end, altered RSV-mediated responses of the innate and adaptive immune system may affect and suppress the generation of a proper immunological memory leading to impaired and short-lived immunity. Comprehending these alterations is thereby of great interest, although further studies will be necessary to address this in more detail.

Solving the prefusion conformation of the RSV F protein and its stabilization demonstrated the immense progress made in vaccine development after decades of basic research. As a consequence of this breakthrough, two preF-based vaccines have recently received licensure for use in older adults as both safety and efficacy were demonstrated. Although the recently developed RSV vaccines seem highly efficacious in older adults, their transmission-reducing potential and durability of protection in this age group require further investigation. The risk of waning antibody levels, also seen after RSV infection, may call for annual vaccination to protect this vulnerable age group.

A preF-protein-based vaccine for the vaccination of pregnant women was licensed recently by the FDA. This way, infants can be protected by vaccine-induced maternally derived antibodies during their first RSV season. Furthermore, several vaccine candidates are currently in phase III clinical trials showing promising results for the vaccination of older adults and pregnant women. Maternal vaccination seems to be a promising approach for the infant population, at least during their first RSV season, by the transplacental transfer of neutralizing antibodies, as direct vaccination of newborns may result in a suboptimal immune response considering their immature immune system. Maternal vaccination, however, may bear certain risks, taking into account the current developments in relation to the RSVPreF3 vaccine and the higher abundance of premature births after vaccination with one candidate vaccine. 

Although a significant amount of data has been collected recently, our understanding of the immunological mechanisms during a natural RSV infection is still incomplete. Considering the phenomena of immunosenescence and preexisting immunity of older adults on one side and the immaturity of the infant’s immune system on the other, analyzing the differences between the immune responses of the two main age groups at risk is critical for vaccine development. Further studies in infants and older adults will be necessary to fully comprehend the protective and harmful immune responses upon RSV infection and vaccination and to pave the way toward a better understanding of cell- and antibody-mediated immunity to RSV infection.
